# Radiomics model to classify mammary masses using breast DCE-MRI compared to the BI-RADS classification performance

**DOI:** 10.1186/s13244-023-01404-x

**Published:** 2023-04-13

**Authors:** Kawtar Debbi, Paul Habert, Anaïs Grob, Anderson Loundou, Pascale Siles, Axel Bartoli, Alexis Jacquier

**Affiliations:** 1 Service de Radiologie, La Timone Hôpital, 264 Rue Saint Pierre, 13005 Marseille, France; 2 Service de Radiologie, Hôpital Nord, Chemin des Bourrely, 13015 Marseille, France; 3 LIIE, Aix Marseille Université, Marseille, France; 4 CERIMED, Aix Marseille Université, Marseille, France; 5 CEReSS UR3279-Health Service Research and Quality of Life Center, Aix-Marseille Université, Marseille, France; 6 Department of Public Health, Assistance Publique - Hôpitaux de Marseille, Marseille, France; 7 UMR 7339, CNRS, CRMBM-CEMEREM (Centre de Résonance Magnétique Biologique et Médicale – Centre d’Exploration Métaboliques par Résonance Magnétique), Assistance Publique - Hôpitaux de Marseille, Aix-Marseille Université, 13385 Marseille, France

**Keywords:** Breast neoplasms, Magnetic resonance imaging, Radiomics, Artificial intelligence

## Abstract

**Background:**

Recent advanced in radiomics analysis could help to identify breast cancer among benign mammary masses. The aim was to create a radiomics signature using breast DCE-MRI extracted features to classify tumors and to compare the performances with the BI-RADS classification.

**Material and methods:**

From September 2017 to December 2019 images, exams and records from consecutive patients with mammary masses on breast DCE-MRI and available histology from one center were retrospectively reviewed (79 patients, 97 masses). Exclusion criterion was malignant uncertainty. The tumors were split in a train-set (70%) and a test-set (30%). From 14 kinetics maps, 89 radiomics features were extracted, for a total of 1246 features per tumor. Feature selection was made using Boruta algorithm, to train a random forest algorithm on the train-set. BI-RADS classification was recorded from two radiologists.

**Results:**

Seventy-seven patients were analyzed with 94 tumors, (71 malignant, 23 benign). Over 1246 features, 17 were selected from eight kinetic maps. On the test-set, the model reaches an AUC = 0.94 95 CI [0.85–1.00] and a specificity of 33% 95 CI [10–70]. There were 43/94 (46%) lesions BI-RADS4 (4a = 12/94 (13%); 4b = 9/94 (10%); and 4c = 22/94 (23%)). The BI-RADS score reached an AUC = 0.84 95 CI [0.73–0.95] and a specificity of 17% 95 CI [3–56]. There was no significant difference between the ROC curves for the model or the BI-RADS score (*p* = 0.19).

**Conclusion:**

A radiomics signature from features extracted using breast DCE-MRI can reach an AUC of 0.94 on a test-set and could provide as good results as BI-RADS to classify mammary masses.

**Supplementary Information:**

The online version contains supplementary material available at 10.1186/s13244-023-01404-x.

## Key points

- The semi-automated breast tumor segmentation method allows extraction of radiomic features.
- A radiomics signature could be extracted from breast DCE-MRI and reach an AUC of 0.94 95%CI [0.85–1.00] on a test-set.
- There was no significant difference between the AUC ROC curves for the model (0.94) or the BI-RADS MRI (0.84) score (*p* = 0.19).

**Supplementary Information:**

The online version contains supplementary material available at 10.1186/s13244-023-01404-x.

## Background

Breast cancer is the most widespread cancer affecting women worldwide with around 2 million cases diagnosed each year [1]*.* A breast MRI is indicated as a second line of imaging because of a high negative predictive value in the detection of malignant lesions [2]. Breast MRI is recommended in patients with a high-risk of cancer, presenting with a high-risk genetic mutation (BRCA-1, BRCA-2, and TP53), and for those with very dense breasts or in case of discordance between the clinical and radiological signs [3]. The main limitation of MRI is its low specificity in the discrimination between benign and malignant lesions, which varies between 47 and 97% according to the literature [4]. This leads to complementary examinations (second-look ultrasound and complementary mammography) and a significant number of biopsies of benign lesions. However, breast MRI is currently the imaging technique that provides the best decision-making performance in the characterization of a benign or malignant lesion based on the BI-RADS criteria [3, 5], but these criteria have a significant degree of inter-observer variability [6].

Radiomics applied to MRI can be defined as a quantitative measurement of the texture parameters extracted from radiological images. These parameters correspond to mathematical descriptors characterizing the shape and heterogenicity of a tumor to a level that is not visible to the naked eye [7]. Radiomics seems to emerge as a new tumoral biomarker for histological or molecular heterogeneity. It could be used to predict the biological nature of a tissue, its therapeutical response or the prognosis for a tumoral lesion [8, 9].

Previous studies have shown the promising results of radiomics in breast MRI in the evaluation of the tumoral response under neoadjuvant chemotherapy, or in the prediction of a histological sub-type of cancer [10, 11], or a molecular sub-type [12]. Other studies have investigated the risk factors of over-expression of the estrogen receptor [13], and lastly others looked into a prognostic analysis linking genomics and radiomics [14]. More recently, studies have used radiomics in the characterization of a benign or malignant lesion by multiparametric MRI with diffusion and perfusion sequences [15], also with high-resolution sequences [16]. Few of these studies concerned standard MRI protocols commonly used to diagnose breast masses (T1-weigthed, T2-weigthed, and dynamic contrast enhancement).

The goals of this study were (1) to develop a new radiomics model suitable for breast MRI to characterize mammary masses, (2) to compare the performance of this model with the BI-RADS classification using histology as gold standard.

## Methods

### Population

**Fig. 1:**
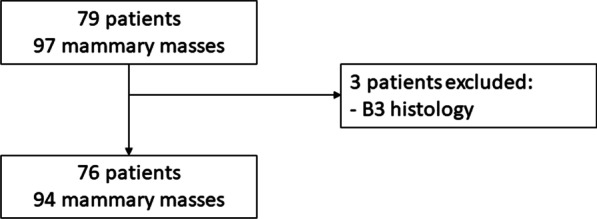
Flowchart of the study

This study is a single-center retrospective study carried out in the radiology department of La Timone University Hospital (Marseille–France). All consecutive patients who had a breast MRI between September 2017 and December 2019 and presented with a mammary mass and histological documentation available were included in the study. According to the BI-RADS classification, a mass is defined as a lesion occupying a volume that is round, oval, or irregular in shape in all three anatomical planes (with convex edges) and visible on the T1-weighted and T2-weighted anatomical sequences. Seventy-nine patients were included, accounting for 97 masses. Three of these mammary masses, histologically classified B3, meaning borderline lesions that were uncertain to be malignant, were excluded. Seventy-six patients for a total of 94 masses were analyzed (Fig. 1). The study was approved by the institutional review board (Comité d’Ethique pour la Recherche en Imagerie Médicale n°CRM-2106-171).

### MRI acquisition parameters

All the patients had breast MRI on the same MR device (Ingenia 1.5 T, Philips medical imaging, Best, the Netherlands). The rationale is detailed in Additional file 1: Appendix 1. The DCE sequences were performed before, then 1, 2, 3, 4, and 5 min after intra-venous injection of gadolinium (DOTAREM: 0.2 cc/kg, Guerbet, Aulnay sous Bois, France). Native reconstructions were performed for each acquisition time (DCE_n_ 0, DCE_n_ 1, DCE_n_ 2, DCE_n_ 3, DCE_n_ 4, and DCE_n_ 5). Subtractions were also made between the post-contrast and pre-contrast acquisitions, and the pre-contrast acquisition was used as a mask for each time-point (DCE_s_ 1, DCE_s_ 2, DCE_s_ 3, DCE_s_ 4, and DCE_s_ 5). The native T1-weighted, T2-weighted, DCE_n_, and DCE_s_ sequences were used for the segmentations of the lesions and analysis performed by the radiologists.

### Data processing (image processing)

**Fig. 2:**
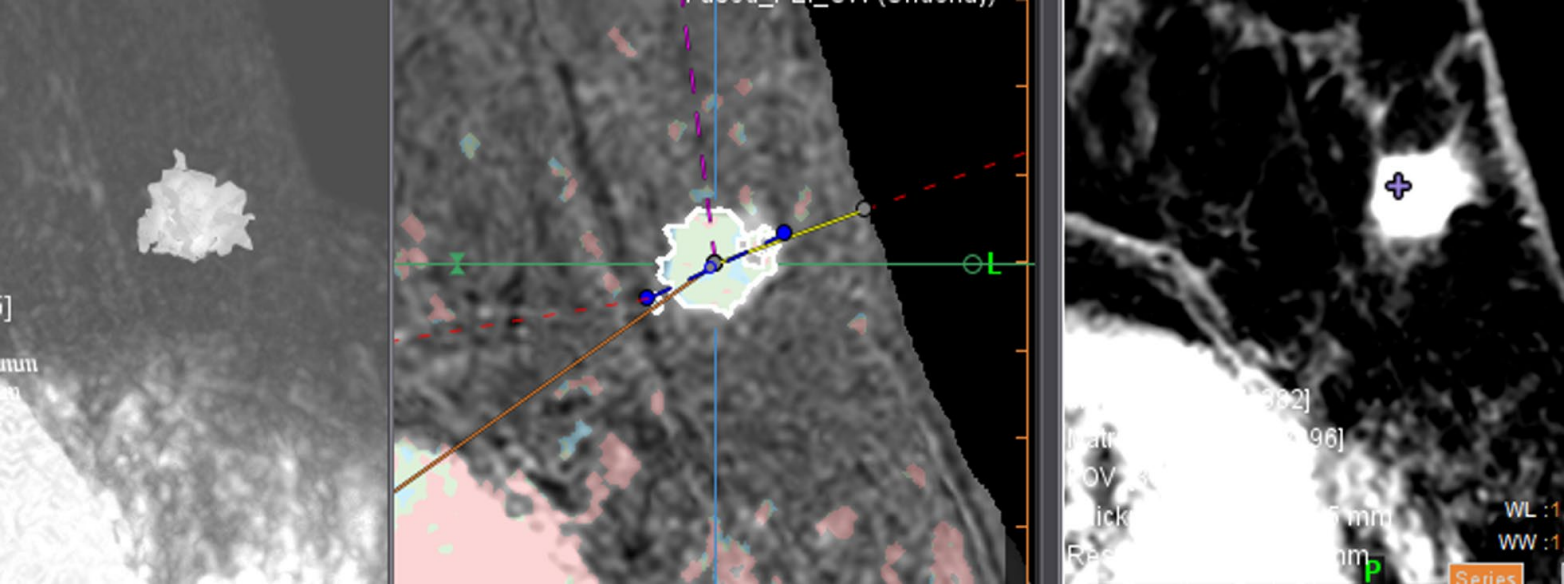
Example of semi-automated segmentation in the axial plane for a grade II infiltrating carcinoma of the left breast on dynamic contrast enhancement subtraction with MIP reconstruction; dynamic contrast enhancement sequence merged with the PEI map; and dynamic contrast enhancement without subtraction

**Fig. 3:**
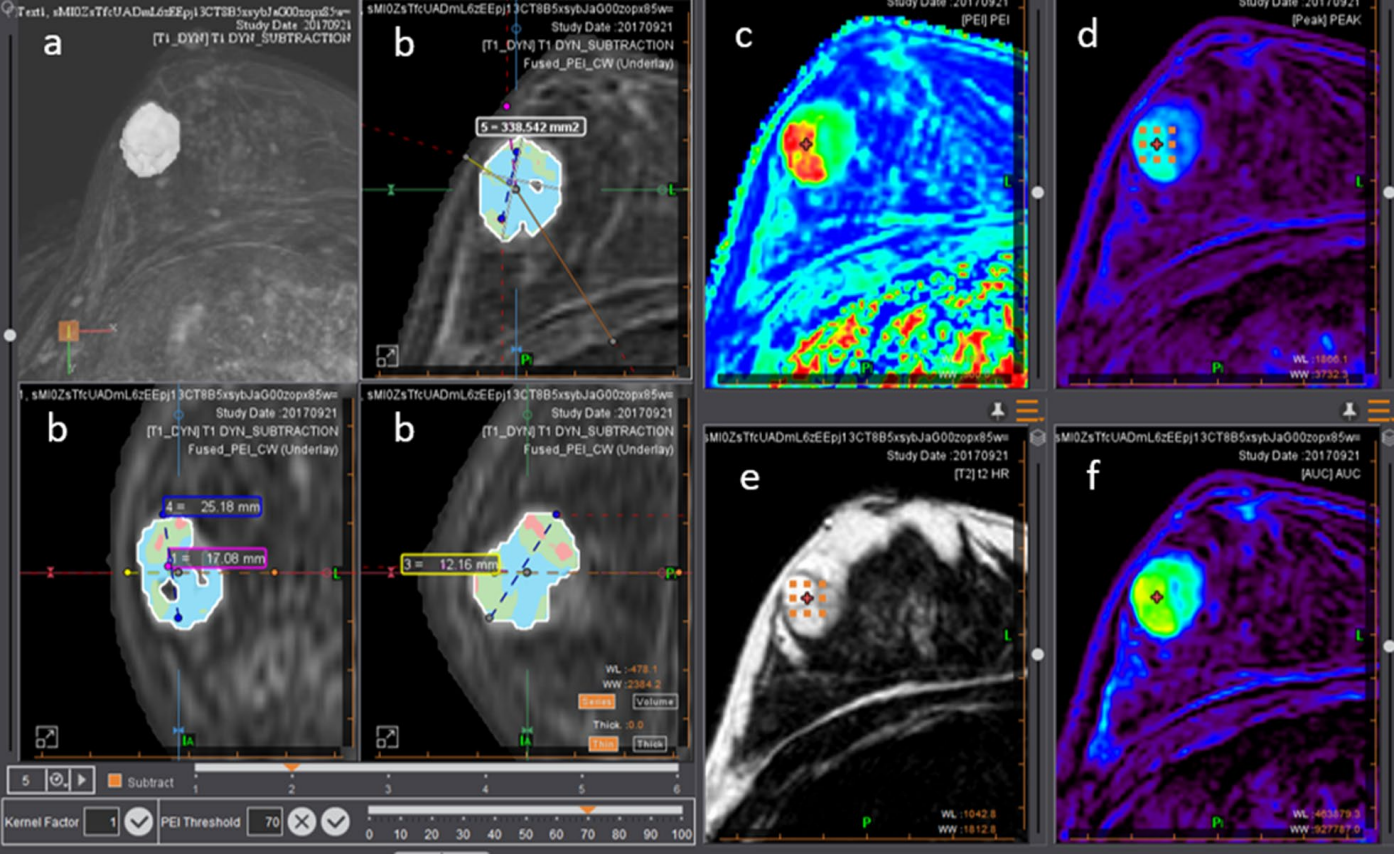
Example of a fibroadenoma at the junction of the external quadrants of the right breast on the breastscape^®^ segmentation software: **a** Dynamic contrast enhancement subtraction with MIP reconstruction; **b** Dynamic contrast enhancement sequence merged with the PEI map in the axial, coronal, and sagittal planes; **c** PEI map; **d** PEAK map; **e** T2-weighted sequence in axial view; **f** AUC map

**Fig. 4:**
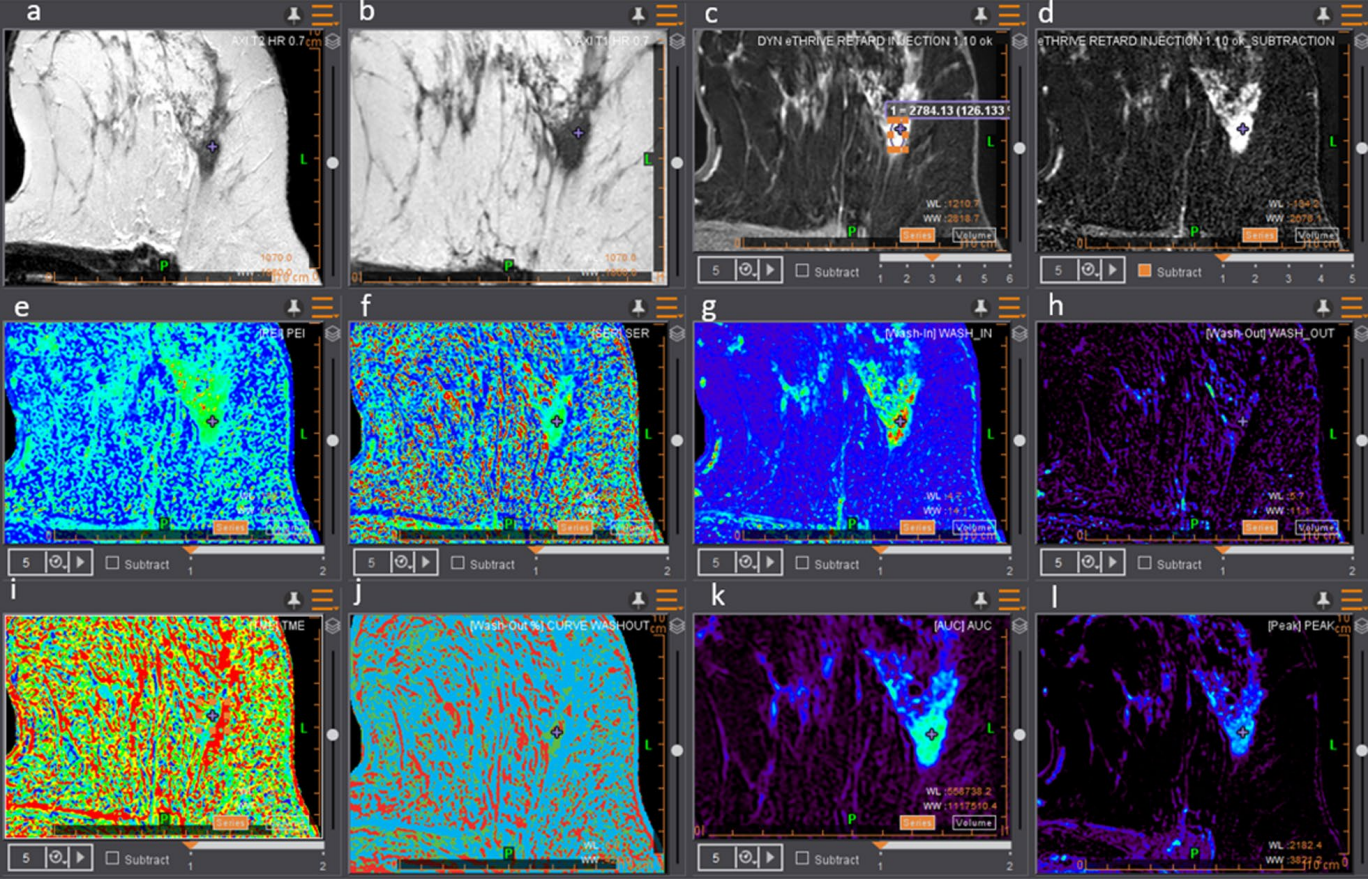
Example of a grade II infiltrating carcinoma of the left breast at the meeting point of the lateral quadrants: **a** T2-weighted sequence in axial view, **b** T1-weighted sequence in axial view, **c** Dynamic contrast enhancement 3 native, **d** Dynamic contrast enhancement 1 subtracted, **e** PEI map, **f** signal enhancement ratio map, **g** WASH IN map, **h** WASH OUT map, **i** TME map, **j** WASHOUT CURVE map, **k** AUC map, **l** Peak map

The images were post-processed using the breastscape^®^ software package (Olea Medical, La Ciotat, France). The masses were segmented semi-automatically on the DCE_s_, after analysis of the whole set of series in the protocols T1-weighted, T2-weighted, and DCE_n_. The DCE_s_ series corresponding to the peak signal was used as a reference series for the segmentation. The segmentation of the lesions proposed in breastscape^®^ enabled us to define the regions of interest (ROI) which corresponded to the intra-mammary mass(es) (Figs. 2, 3 and 4). No image preprocessing technique, such as discretization of the images before calculation of the radiomics parameters, was used. Whenever necessary, the motion was corrected on the dynamic sequences [17].

### Extracting the parameters

**Fig. 5:**
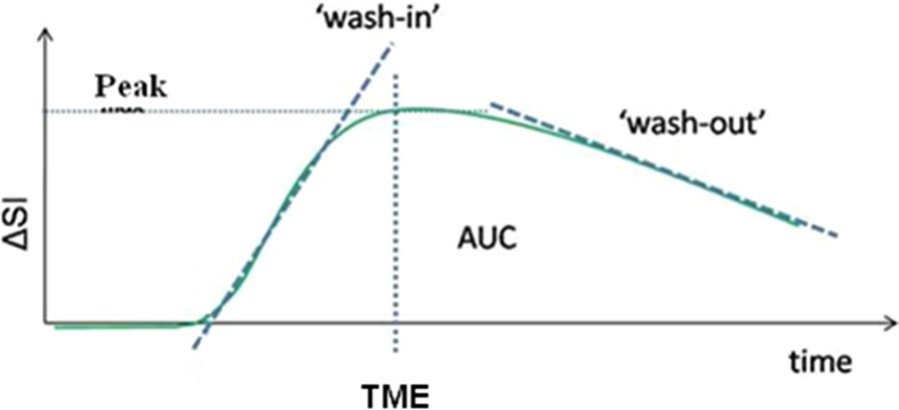
Signal intensity curve over time

Texture parameters were extracted from the different series: DCE_s_ (DCE_s_ 1, DCE_s_ 2, DCE_s_ 3, DCE_s_ 4, and DCE_s_ 5) and from eight maps based on signal enhancement values calculated by the breastscape^®^ software package referred to as *kinetics* by the software (Fig. 5). Details concerning the calculation of the kinetics maps are available in Additional file 1: Appendix 2. The texture parameters were extracted using the pyradiomics library (https://pyradiomics.readthedocs.io/en/latest/). Based on this library, an executable parallel code, called breast features, has been developed to extract the texture parameters available via Pyradiomics, using the semi-automatic segmentation as mask.

The texture descriptors are separated into three groups: the shape, the first order, and the texture descriptors. Shape descriptors refer to the contours and the morphology of the lesion and to its size. Descriptors of the first-order describe the distribution of intensity and levels of gray in the pixels or voxels based on a histogram that shows the distribution of the different parameters of the signal. Texture parameters of the second order describe the matrix of the different parameters of distribution of pixels in the image. The list of the different parameters extracted is available in Additional file 1: Appendix 3.

### BI-RADS analysis

Two senior radiologists specialized in breast imagining (P.S. 10-year experience, A.G. 5-year experience) classified the mammary masses on breast MRI according to the BI-RADS classification; a mass classified BI-RADS 2 or 3 was considered as benign or very probably benign and masses classified BI-RADS 4 or 5 as highly suspected of malignancy [18, 19] (Additional file 1: Appendix 4).

### Histological analysis

The histological analysis was used as a diagnostic reference. An anatomopathological analysis was established by ultrasound-guided needle microbiopsy using a 14 Gauge needle or by analyzing the fragment obtained after tumor resection. To assess the pathologic-imaging concordance, a clip was deployed if the lesion size was < 1.5 cm or if the lesion had become no invisible immediately after biopsy. A mammary MRI was performed after clip deployment in cases of neoadjuvant chemotherapy, multiple lesions, or extreme fibroglandular tissue. The histological results were classified according to the European classification: B2 for benign lesions and B5 for malignant lesions. All benign lesion were controlled using ultrasound 3 months after the biopsy to ensure the non-malignancy.

### Statistical analysis

Continuous data with a normal distribution are expressed as the mean ± standard deviation. Categorical data are expressed as frequencies or percentages. Pyradiomics extracted 89 features per tumor from 14 kinetics maps, for a total of 1246 features per tumor. The whole dataset comprising 94 masses was split in a train-set (70%) and a test-set (30%) with stratification on the histology. On the train-set, Boruta’s algorithm was used to select the most relevant descriptors among the 1246 extracted [20, 21]. The importance of each descriptor was calculated by 99 iterations which generated a mean importance value; the higher the score, the more important the descriptor. The algorithm classified the descriptors according to three types: (1) confirmed, indefinite, or non-confirmed discriminatory descriptors. A random forest algorithm was used as the model, trained on the train-set and then applied on the test-set. The performances of the model included the ROC parameters: the area under the curve (AUC), the accuracy, sensitivity, specificity, according to a confidence interval (CI) at 95% for each dataset. The Bootstrap test compared the AUCs and the ROC curves. All the statistical analyses were performed using the R software package (version 4.1.0). A significant difference was obtained for a *p*-value < 0.05.

## Results

### Histological data

Seventy-six patients were included, accounting for 94 masses. Seventy-one (75.5%) masses were malignant, and 23 (24.5%) were benign. Among the malignant lesions, there were 3/71 (4.2%) infiltrating lobular carcinomas and 68/71 (95.8%) non-specific infiltrating carcinomas. Among the benign lesions, there were 7/23 (30.4%) fibroadenomas; 5/23 (21.7%) ductal ectasia; 5/23 (21.7%) adenosis or fibrotic lesions; 3/23 (13.0%) of ductal cysts; 2/23 (8.7%) cytosteatonecrotic lesions; and 1/23 (4.4%) abscess (Table 1). The median lesion size was 24 mm (IQR = 47 mm). None of the 23 benign lesions had grown 3 months after the biopsy.

**Table 1:** Histological results of the masses studied

Type of tumor	*N* = 94
Benign	23
Fibroadenoma	7 (30)
Duct ectasia	5 (22)
Adenosis, fibrosis	5 (22)
Duct cyst	3 (13)
Cytosteatonecrosis	2 (9)
Abscess	1 (4)
Malignant	71
Non-specific infiltrating carcinoma	68 (96)
Infiltrating lobular carcinoma	3 (4)

Data are given as number (percentages)

### Selected features

Out of the 1246 descriptors, 1228 were non-confirmed, 6 were confirmed and 12 were considered as indefinite. We decided to keep the 6 that were confirmed and the 12 indefinites for a total of 18 features, to make a predictive model. The radiomics signature contained: the “inverse difference moment normalized” (IDMN), “inverse difference normalized” (IDN), “low gray run emphasis” (LGRE), “long run low gray level emphasis” (LRLGLE), “short run low gray level emphasis” (SRLGLE), “informal measure of correlation” (IMC), “large area low gray level emphasis” (LALGLE), “long run high gray level emphasis” (LRHGLE), “maximum 3D diameter,” “total energy, major axis,” and “energy.” Only the subtracted dynamic maps DCE 1_ s_, DCE_s_ 2, DCE_s_ 4, and DCE_s_ 5 and the *kinetics* maps AUC, peak enhancement, signal enhancement ratio, and washout contained at least one descriptor that was useful for the creation of the model. Among the 18 descriptors retained by the Boruta method, one descriptor was excluded because the coefficient of importance was equal to zero. The model was finally created integrating 17 discriminatory descriptors: four were shape variables, two variables of the first order, and 11 variables of the second order.

Some descriptors were found several times in different maps or sequences, such as the shape descriptor «maximum 3D Diameter» or one of the second-order descriptors such as IDN and IDNM. IDNM was discriminatory for the DCE 1 and DCE 2 sequences. The descriptors that enabled us to create the predictive model are given in Table 2.

**Table 2:** Descriptors retained for the creation of the model to predict malignant or benign masses

Type of dynamic MRI card	Shape features	First-order features	Second-order features	Importance coefficient
DCE1			Inverse difference normalized	97.9
			Inverse difference moment normalized	62.3
DCE2	Maximum 3D diameter			18.3
			Inverse difference normalized	91.9
			Inverse difference moment normalized	100.0
DCE4	Maximum 3D diameter			13.1
DCE5			Informal measure of correlation 1	46.3
AUC			Low gray level run emphasis	67.2
			Long run low gray level run emphasis	59.4
			Short run low gray level run emphasis	53.5
			Inverse difference moment normalized	40.7
			Long run high gray level run emphasis	33.9
PE			Long run high gray level emphasis	32.2
SER		Total energy		13.3
		Energy		4.4
WO	Major axis			7.2
	Maximum 3D diameter			20.6

*AUC* Area under curve; *DCE* Dynamic contrast enhancement; *PE* Peak enhancement; *SER* Signal enhancement ratio; *WO* Washout

### Diagnostic performances of the radiomics predictive model and the BI-RADS classification

The sensitivity of the model in characterization of malignant lesion was 100% (95% CI [84.5‒100.0]) with a specificity of 33.3% (95% CI [9.7‒70.0]). The accuracy of the diagnosis was 85% (95% CI [66.3‒95.8]). The area under the curve (AUC) based on the test sample is 0.94 (95% CI [0.85‒1.00]) (Table 3).

**Table 3:** Diagnostic performances of the radiomic predictive model and the BI-RADS analysis carried out by the radiologist

	Radiomics model performance	BI-RADS performance
Sensibility	100% [85–100]	100% [95–100]
Specificity	33% [10–70]	17% [3–56]
Positive predictive value	84% [65–94]	80% [70–87]
Negative predictive value	100% [34–100]	100% [57–100]
Accuracy	0.85 [0.66–0.96]	0.82 [0.63–0.92]
AUC	0.94 [0.85–1]	0.84 [0.73–0.95]

**Fig. 6:**
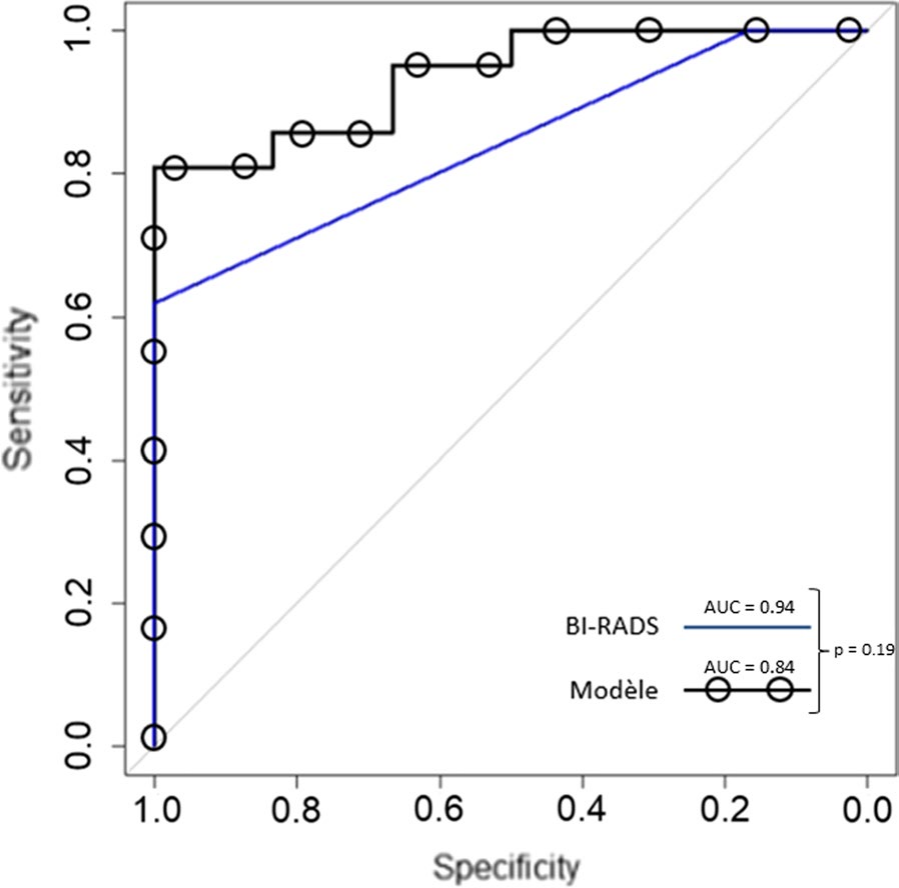
ROC curve of the BI-RADS score (continuous curve) and of the radiomics model (curve with circles)

Almost half 46/94 (48.9%) of the mammary masses were classified BI-RADS 5, typically malignant. There were only five masses classified typically benign or probably benign. The BI-RADS 4c included 22/94 (23.4%) tumors and 9/94 (9.6%) lesions BI-RADS 4b (Table 4). According to the BI-RADS criteria used by the radiologists, the sensitivity was 100% (95% CI [84.5, 100]), and the specificity was 16.7% (95% CI [3.0, 56.4]). The accuracy of the diagnosis was 81.5% (95% CI [63.3, 91.8]). The AUC was 0.84 (95% CI [0.73, 0.95]). The AUC of the model tended toward a relatively higher score than for BI-RADS, 0.94 versus 0.84 (*p* = 0.19) without significant difference (Fig. 6).

**Table 4:** BI-RADS analysis

BI-RADS score	*N* = 94	Malignant histology	Benign histology
2	1 (1.0)	0 (0%)	1 (100%)
3	4 (4.3)	1 (25%)	3 (75%)
4a	12 (12.8)	5 (42%)	7 (58%)
4b	9 (9.6)	6 (66%)	3 (34%)
4c	22 (23.4)	12 (55%)	10 (45%)
5	46 (48.9)	45 (98%)	1 (2%)

Data are given as number (percentages)

## Discussion

That study enabled us to create a predictive model to characterize mammary masses as benign or malignant with a high AUC = 0.94 on a test-set. This level of AUC was not different from that of two experienced radiologists based on the BI-RADS criteria. There seems to be an improvement in specificity in comparison with BI-RADS (33.0% (95% CI [9.7–70] against 16.7% (95% CI [3.0–56.4]) although the confidence intervals overlap.

The number of malignant lesions was superior to the number of benign lesion because the latter are less frequently biopsied. The fact that benign lesions are found in the histological results shows quite clearly that too many histological samples are taken because of the low specificity of the methods used in current practice. It has been demonstrated that the morphological parameters such as shape and outline are essential on MRI for an accurate diagnosis of breast cancer, based on the BI-RADS criteria [22]. But there is a high degree of inter-observer variability [6]. These shape and outline parameters that are present in the BI-RADS lexicon are also modeled in the shape descriptors in radiomics, such as elongation and sphericity. However, in this case, these were not the most significant variables retained by the selection algorithm. The only discriminatory morphological variables retained were the maximum 3D diameter and the major axis.

The final statistical model retained mainly descriptors of the second order (11/17). Some texture descriptors are present in several signal enhancement maps or series of dynamic images such as for IDN and DMN which have the highest coefficient of importance. In addition, their discriminatory nature is present in early DCE_s_ (DCE_s_ 1 and DCE_s_ 2). Fusco et al. have already demonstrated the relationship between the kinetics maps of a lesion and its histological prognosis [23]. Many recent studies have been focused mainly on the early acquisition times in characterizing a malignant lesion as shown in Vande Perre et al. study on the characterization of a malignant or benign lesion at an early stage of injection [16]. Malignant and benign tumors do not enhance in the same way. Even if the enhancement curves overlap, a benign lesion will enhance gradually (type III curve) [24]*.* This is explained by the neo-angiogenesis in malignant tumors and by an increase in capillary patency. These details apply mainly to infiltrating carcinoma with no specificity, the predominant malignant histology in this study. Compared to infiltrating carcinoma, lobular carcinoma and ductal carcinoma show a later enhancement [25]. This highlights the relevance of dynamic analysis of the texture parameters [26]. Recent studies have looked into descriptors for perfusion-MRI that would be more representative of tumoral capillary patency [15].

Another application of radiomics could be the prediction of molecular sub-type and androgen receptor expression using breast MRI. A recent study on 162 patients showed the ability of multiparametric breast MRI to discriminate androgen receptor expression and molecular sub-types (AUC = 0.907 and 0.965, respectively) using the multilayer perceptron algorithm which performed slightly better than the random forest algorithm in their population (AUC = 0.905 and 0.897, respectively) [27].

The parameters that are not visible to the naked eye could be a major asset for the radiologists. In breast MRI, machine learning attempts to combine human interpretation based on the BI-RADS criteria and the radiologist’s knowledge with the data of multiparametric imaging. Despite the large number of studies on the topic, a lack of homogeneity in the data extraction and texture analysis are strong limits to use these algorithms into practice. The method used in this study has already been described in the literature [28].

Acquisitions were performed according to a standard rationale including morphological and dynamic sequences which came from the same center. We used a semi-automated segmentation software package (breastscape^®^, Olea Medical, La Ciotat, France). This technique has the advantage of having better reproducibility in the segmentation of texture parameters than manual segmentation [29].

Among all the texture parameters that exist in the literature, none alone can discriminate a lesion. The final statistical model contains only a few descriptors compared to all the variables tested initially (17 out of 1246 features), thanks to the exclusion of the redundant and non-reproducible parameters. We adapted the number of parameters to the number of lesions analyzed to reduce overfitting by machine learning [30].

Previous studies have already proven that machine learning algorithms could be successfully applied in breast MRI [31] with the possibility of generating interesting results but very few studies have reached an AUC of 0.94 [32]. The study of Nie et al. was based on the same type of study with an AUC at 0.82 but the number of descriptors analyzed was much lower.

This MRI rationales included a morphological T2 sequence to confirm the nature of the mass and to help to establish the contours of the ROI. But the data analysis was based only on the enhancement sequences. This aspect is interesting with the increasing development of fast sequences aimed at reducing the breast MRI protocols, using in some cases only the injected sequences [33, 34]. Conversely, some studies have shown that adding extra, combined sequences adds more overall accuracy in the discrimination between benign/malignant tumors, in particular in the case of diffusion sequences and the calculation of ADC which improves the specificity of the discrimination between a malignant and a benign tumor [35]. In Zhang et al. study, the diagnostic performances of the multiparametric model had an AUC at 0.92 against 0.84 when only injected sequences were used [15].

This study suffer from some limitations; the main is the lack of evaluation of the problematic subgroups: BI-RADS IVa and IVb masses. Theses subgroups were too small to be analyzed with radiomics. This should be the main target of this model, in clinical practice. Characterizing BI-RADS IVc and BI-RADS V masses with this radiomics model has a low clinical impact for trained breast radiologists. Similarly, the lack of evaluation of this radiomics model in case of small masses (< 10 mm) is another main limitation to clinical practice. A third limitation is the lack of external validation on independent cohorts collected from other centers [36]. This limitation of clinical application is linked to the difficulty in obtaining large cohorts of patients in each center. Breast MRI is often carried out as a second line examination after the patient has been screened by mammography and not all mammary masses are sampled. In addition, non-mass enhancements were not taken into account in this study which was based only on lesions that were masses, keeping in mind the fact that many cancerous lesions are enhanced as non-mass [37].

## Conclusion

This single-center study enabled us to create a predictive algorithm based on radiomics to predict breast masses as benign or malignant with good performances. The predictive model yields performances equivalent to those analyzed using the BI-RADS criteria, with an AUC at 0.94 (95% CI [0.85–1.00]) on a test-set. To improve the specificity of the BI-RADS criteria, this model could be a major asset for clinical practice, but the model needs to be evaluated on the BI-RADS IVa and IVb lesions in the future as these are the problematic categories in clinical practice. Multi-center studies with external datasets could allow us to assess whether this type of approach would decrease the number of unnecessary biopsies.

## Supplementary Information

**Additional file 1.** Supplementary details on the MRI sequence, MAPS, radiomics features and BI-RADS MRI Lexicon.

## Data Availability

Data are available upon request.

## Abbreviations

AUC: Area under the curve
CW: Curve washout
DCE: Dynamic contrast enhancement
FA: Flip angle
FOV: Field of view
IDMN: Inverse difference moment normalized
IDN: Inverse difference normalized
IMC: Informal measure of correlation
LALGLE: Large area low gray level emphasis
LGRE: Low gray run emphasis
LRHGLE: Long run high gray level emphasis
LRLGLE: Long run low gray level emphasis
PE: Peak enhancement
PEI: Peak enhancement intensity
SER: Signal enhancement ratio
SRLGLE: Short run low gray level emphasis
TE: Echo time
TME: Time to maximal enhancement
TR: Repetition time
WI: Wash in
WO: Wash out
