# Supplementary material for: Radiomics model to classify mammary masses using breast DCE-MRI compared to the BI-RADS classification performance
Source: Insights Imaging. 2023 Apr 13;14:64. doi: 10.1186/s13244-023-01404-x (PMC10102264; doi:10.1186/s13244-023-01404-x)
Supplement: Supplementary file 1 — Additional file 1. Supplementary details on the MRI sequence, MAPS, radiomics features and BI-RADS MRI Lexicon. [file 13244_2023_1404_MOESM1_ESM.pdf]

ELECTRONIC SUPPLEMENTARY MATERIAL

Radiomics model to classify mammary masses using breast DCE-MRI compared to the BI-RADS classification performance

Appendix 1

T1: relaxation time (TR) = 441.5 msec, echo time (TE) = 9.0 msec, acquisition matrix = 544 x 538, reconstruction matrix = 768 x 768, field of view (FOV) = 38 x 38 cm2, falling angle (FA) = 90°, slice thickness = 3.3mm. T2: TR = 4609.6 msec, TE = 120.0 msec, acquisition matrix = 544x505, reconstruction matrix =768 x 768, FOV =38 x 38 cm2, FA= 90°, slice thickness= 3.3mm. Dynamic contrast enhanced (DCE): TR = 5.7 msec, TE = 2.8 msec, acquisition matrix = 424x382, reconstruction matrix = 896x896, FOV = 36x36cm2, FA = 12° slice thickness = 1.5 mm.

Appendix 2

1 – Maps based on the absolute enhancement of the signal:

Figure 1 shows the signal intensity curve over the period required to calculate the maps based on absolute enhancement of the signal.

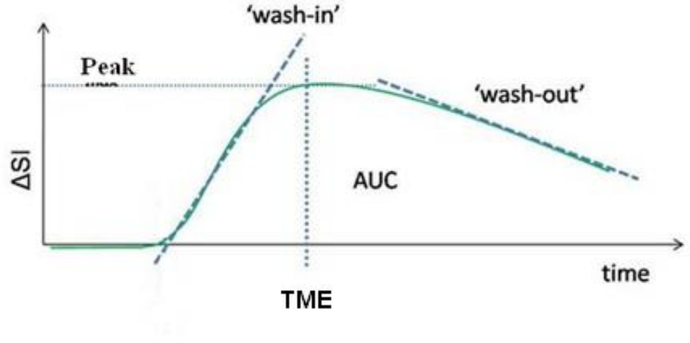

Figure 1 : signal intensity curve in time.

Time to maximum enhancement (TME): the TME map shows the regional distribution of time required for the increase in signal to reach its peak value.

Area under the curve (AUC): this map shows the integration of the signal increase curve over a period in time.

PEAK enhancement (P): this map shows the peak enhancement of the signal over a period of time.

Initial ascending slope (WASHIN): this map shows the initial ascending slope of the signal increase curve in relation to time.

Descending slope (WASHOUT): this map shows the descending slope of the signal curve in time between the peak phase and the last time-point on the curve.

2 – Maps based on the relative enhancement of the signal:

Three time-points based on the dynamic enhancement sequences are necessary to calculate the curves based on the relative enhancement of the signal: the mask time-point (t0), the end of the wash-in phase, the peak of the signal (t1) and the last phase (t2). S0, S1 and S2 correspond to the intensity of the signal at the three time-points.

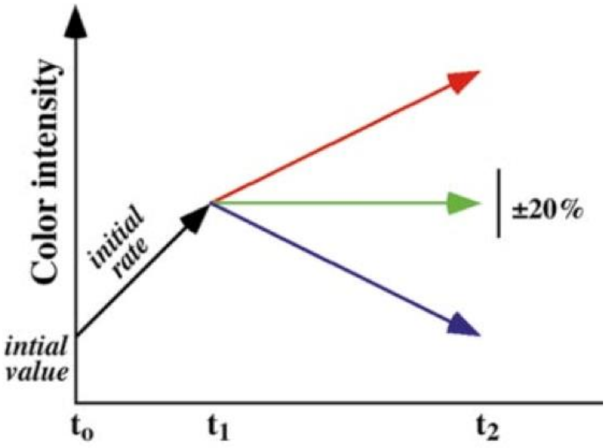

Figure 2 : three time-points in signal intensity allow the calculation of maps based on the relative enhancement of the signal.

From these three time-points the breastscape® software package calculated four descriptive parameters that are:

Peak Enhancement Intensity (PEI): this map represents the initial maximum intensity of the signal curve in time *****. $\mathbf{100\ x\ \frac{S1-S0}{S0}}$

Relative wash out (CURVE WASHOUT): this map shows the percentage of descending slope of the signal enhancement curve v $\mathbf{100\ x\ \frac{S2-S1}{S1}}$

Signal Enhancement Ratio (SER): this map shows the signal enhancement ratio on the signal enhancement curve in time. $\mathbf{100\ x\ \frac{S1-S0}{S2-S0}}$

Appendix 3

Forme	1er ordre	2nd ordre
Elongation	10 Percentile	Autocorrelation
Flatness	90 Percentile	Cluster Prominence
Least Axis Length	Energy	Cluster Shade
Major Axis Length	Entropy	Cluster Tendency
Maximum 2D Diameter Column	InterquartileRange	Contrast
Maximum 2D Diameter Row	Kurtosis	Correlation
Maximum2D Diameter Slice	Maximum	Difference Average
Maximum 3D Diameter	MeanAbsoluteDeviation	Difference Entropy
Mesh Volume	Mean	Difference Variance
Minor Axis Length	Median	Inverse Difference (ID)
Surface Volume Ratio	Minimum	Inverse Difference Moment (IDM)
Voxel Volume	Range	Inverse Difference Moment Normalized (IDMN)
Compactness 1	Root Mean Squared	Inverse Difference Normelized (IDN)
Compactness 2	Skewness	Informal Measure of Correlation 1 (IMC 1)
Spherical Disproportion	Total Energy	Informal Measure of Correlation 2 (IMC2)
	Uniformity	Inverse Variance
	Variance	Joint Average
	Standard Deviation	Joint Energy
		Joint Entropy
		Maximal Correlation Coefficient (MCC)
		Maximum Probability
		Sum Average
		Sum Entropy
		Sum Squares
		Gray Level Non Uniformity
		Gray Level Non Uniformity Normalized
		Gray Level Variance
		High Gray Level Run Emphasis
		Long Run Emphasis
		Long Run High Gray Level Emphasis
		Long Run Low Gray Level Emphasis
		Low Gray Level Run Emphasis
		Run Entropy
		Run Length Non Uniformity
		Run Length Non Uniformity Normalized
		Run Percentage
		Run Variance
		Short Run Emphasis (SRE)
		Short Run High Gray Level Emphasis (SRHGLE)
		Short Run Low Gray Level Emphasis (SRLGLE)
		High Gray Level Zone Emphasis (HGLZE)
		Large Aera Emphasis (LAE)
		Large Aera High Gray Level Emphasis (LAHGLE)
		Large Area Low Gray Level Emphasis (LALGLE)
		Low Gray Level Zone Emphasis (LGLZE)
		Size Zone Non Uniformity (SZNU)
		Size Zone Non Uniformity Normalized (SZNUN)
		Small Area Emphasis (SAE)
		Small Area High Gray Level Emphasis (SAHGLE)
		Small Area Low Gray Level Emphasis (SALGLE)
		Zone Entropy
		Zone Percentage
		Zone Variance

Appendix 4

BI-RADS MRI Lexicon (5th edition)

D'Orsi CJ, Sickles EA, Mendelson EB, Morris EA. ACR BI-RADS Atlas. Breast Imaging Reporting and Data System. Reston, VA:American College of Radiology; 2013.

SHAPE	EDGES	INTERNAL ENHANCEMENT
OVAL	CIRCUMSCRIBED	HOMOGENOUS
ROUND	IRREGULAR	HETEROGENOUS
IRREGULAR	SPICULATED	PERIPHERAL

BI-RADS Analysis Score

BI-RADS *American College of radiology*	Risk of malignancy
1	Assessment normal (VPP 0%)
2	Benign anomalies (VPP 0%)
3	Probably benign (VPP<2%)
4 (a, b, c)	Suspicious (VPP 2 at 95%)
5	Malignant (VPP>95 %)
6	Malignancy proved (biopsy or cyto +)
